# Diagnostic accuracy of handheld fundus photography: A comparative study of three commercially available cameras

**DOI:** 10.1371/journal.pdig.0000131

**Published:** 2022-11-02

**Authors:** Louisa Lu, Somsanguan Ausayakhun, Sakarin Ausayakuhn, Preeyanuch Khunsongkiet, Atitaya Apivatthakakul, Catherine Q. Sun, Tyson N. Kim, Michele Lee, Edmund Tsui, Plern Sutra, Jeremy D. Keenan

**Affiliations:** 1 Francis I. Proctor Foundation, University of California, San Francisco, San Francisco, California, United States of America; 2 Department of Ophthalmology, Stanford University, Stanford, California, United States of America; 3 Department of Ophthalmology, Faculty of Medicine, Chiang Mai University, Chiang Mai, Thailand; 4 CMU Lasik Center, Faculty of Medicine, Chiang Mai University, Chiang Mai, Thailand; 5 Sriphat Medical Center, Faculty of Medicine, Chiang Mai University, Chiang Mai, Thailand; 6 Department of Ophthalmology, University of California, San Francisco, San Francisco, California, United States of America; 7 Department of Ophthalmology, University of Washington, Seattle, Washington, United States of America; The University of Sydney, AUSTRALIA

## Abstract

The objective of this study was to compare the sensitivity and specificity of handheld fundus cameras in detecting diabetic retinopathy (DR), diabetic macular edema (DME), and macular degeneration. Participants in the study, conducted at Maharaj Nakorn Hospital in Northern Thailand between September 2018 and May 2019, underwent an ophthalmologist examination as well as mydriatic fundus photography with three handheld fundus cameras (iNview, Peek Retina, Pictor Plus). Photographs were graded and adjudicated by masked ophthalmologists. Outcome measures included the sensitivity and specificity of each fundus camera for detecting DR, DME, and macular degeneration, relative to ophthalmologist examination. Fundus photographs of 355 eyes from 185 participants were captured with each of the three retinal cameras. Of the 355 eyes, 102 had DR, 71 had DME, and 89 had macular degeneration on ophthalmologist examination. The Pictor Plus was the most sensitive camera for each of the diseases (73–77%) and also achieved relatively high specificity (77–91%). The Peek Retina was the most specific (96–99%), although in part due to its low sensitivity (6–18%). The iNview had slightly lower estimates of sensitivity (55–72%) and specificity (86–90%) compared to the Pictor Plus. These findings demonstrated that the handheld cameras achieved high specificity but variable sensitivities in detecting DR, DME, and macular degeneration. The Pictor Plus, iNview, and Peek Retina would have distinct advantages and disadvantages when applied for utilization in tele-ophthalmology retinal screening programs.

## Author summary

Telemedicine holds much promise to improve health care. One potential implementation of telemedicine is the use of remote diagnostics for screening. A diagnostic test could be performed in the community, or in a primary care clinic, and abnormal test results could trigger referral to the relevant provider. Eye diseases are well-suited to telemedicine approaches given the availability of portable imaging devices. For example, a number of handheld retinal cameras have been developed that could be used to screen for eye diseases like diabetic retinopathy and age-related macular degeneration. However, very few studies have compared different devices to assess their suitability for screening purposes. In this study we compared three portable, handheld retinal cameras. A group of ophthalmologists graded the images for diabetic retinopathy and macular degeneration. The photo-grades were compared with the in-person clinical exam done by another ophthalmologist. We found that one of the cameras, the Pictor Plus, had the best balance of detecting most of the true positive cases while minimizing the number of false positive cases. Although technologies may change, this study provides useful benchmarks to judge the accuracy of handheld retinal cameras.

## Introduction

Diabetic retinopathy (DR) and age-related macular degeneration (AMD) are leading causes of blindness worldwide. Combined, these diseases account for approximately one-third of the blindness in industrialized countries, and are now becoming increasingly prevalent in rapidly developing low- and middle-income countries. [1–4] Both DR and AMD typically have an asymptomatic onset but ultimately can progress to cause irreversible vision loss. [5] Timely screening for these diseases could thus allow for early diagnosis and treatment, especially in areas of the world with a relative shortage of eye care providers and health care resources.[6,7]

Fundus photography has proven to be an effective modality for detecting retinal abnormalities, but the majority of studies in the literature have analyzed photographs from traditional table-mounted retinal cameras. [8–15] Traditional retinal cameras are bulky, expensive (e.g., 2022 cost of a Topcon Retinal Camera is 19900 USD), and require skilled personnel for operation, making them an impractical and cost-prohibitive tool for screening. [16,17] The development of handheld fundus cameras, including devices that are smartphone-based, holds promise to overcome these limitations and enhance opportunities for telemedicine diagnosis of retinal disease. [18]

Several handheld fundus cameras have been previously tested in comparison with traditional retinal cameras or clinical examinations for the screening of retinal diseases such as diabetic retinopathy and for diseases of the optic nerve such as glaucoma and optic neuritis, with generally positive results. [16,18–22] Such handheld cameras could have a variety of applications, including community-based retinal screening, screening at primary care or non-ophthalmologic medical clinics (e.g., endocrinology), and screening or management in remote areas with poor access to eye care services. However, few studies have compared multiple handheld fundus cameras in the same study population, leaving a gap in the literature for a study that adequately assesses the relative merits of different devices. The objective of this study was to compare the diagnostic accuracy of three different handheld fundus cameras versus a reference standard ophthalmologist examination for the diagnosis of diabetic retinopathy and age-related macular degeneration.

## Methods

### Ethics

Approval for this study was obtained from the Institutional Review Board at the University of California, San Francisco and the Chiang Mai University Faculty of Medicine Research Ethics Committee. The research adhered to the tenets of the Declaration of Helsinki. Written informed consent was obtained from all participants.

### Context

The Ministry of Public Health of Thailand has a nationwide diabetic retinopathy screening program in which non-physician healthcare professionals in primary care clinics and community hospitals take fundus photographs using traditional tabletop cameras. As far as we know, the program has not published pre-specified recommendations for diagnostic accuracy for the fundus cameras in the program. The goal of the Ministry of Public Health is to screen 60% of all people with diabetes nationwide. However, a 2017 report revealed that the program was falling short of its target with a coverage rate of around 50% of the population with diabetes. [23]

### Study design and participants

This cross-sectional, observational, single-site instrument validation study was conducted at two outpatient ophthalmology clinics (i.e., a comprehensive clinic and an intraocular injection clinic) at the Department of Ophthalmology of Maharaj Nakorn Hospital in Chiang Mai, Thailand between September 2018 and May 2019. Participants 18 years or older who had been examined by an ophthalmologist at Maharaj Nakorn Hospital during this window were eligible for enrollment. Participants underwent pupil dilation and had fundus photographs taken with three different handheld fundus cameras in random order. The diagnostic accuracy of each camera was assessed relative to the most recent ophthalmologist examination.

### Reference standard examination

All participants underwent a comprehensive dilated slit lamp examination by an ophthalmologist who was also a retina specialist. Fundoscopic exam findings of the optic nerve, macula, and periphery were recorded on a standardized examination form; these forms were reviewed for the presence of diabetic retinopathy (recorded as mild-to-moderate nonproliferative DR [NPDR], severe NPDR, or proliferative DR), diabetic macular edema, age-related macular degeneration, polypoidal choroidal vasculopathy, findings associated with macular degeneration (i.e., choroidal neovascularization and pigment epithelial detachment), and other retinal pathologies.

### Index tests

Participants had a sequence of fundus photographs obtained with each of three different handheld fundus cameras: iNview (Volk, Mentor, OH), Peek Retina (Peek Vision, London, UK), and Pictor Plus (Volk, Mentor, OH). These particular cameras were specifically chosen because they encompassed three different design factors and price points (Table 1). The Pictor Plus is a non-mydriatic handheld fundus camera featuring a handset with a digital ophthalmoscope attachment that provides a 40-degree static field of view (2022 cost: 6800 USD; no smartphone needed). The iNview is a smartphone-based retinal imaging attachment featuring a 20D funduscopic condensing lens that provides a 50-degree static field of view (2022 cost: 800 USD for the device alone; 1000 USD for the device and an iPhone 6). The Peek Retina is a smartphone-based retinal imaging clip-on attachment featuring a prism assembly that provides a 20-degree field of view (2022 cost: 220 USD for the device alone; 420 USD for the device and an iPhone 6). The same Apple iPhone 6 (Apple Inc., USA) was used to capture images with the Volk iNview and the Peek Retina.

**Table 1:** Comparison of characteristics of handheld fundus cameras.

Camera	Features	Field of view	Price (in 2022)
iNview	Smartphone-based retinal imaging attachment featuring a 20D funduscopic condensing lens	50-degree static field of view	800 USD for the device alone; 1000 USD for the device and an iPhone 6
Peek Retina	Smartphone-based retinal imaging clip-on attachment featuring a prism assembly	20-degree static field of view	220 USD for the device alone; 420 USD for the device and an iPhone 6
Pictor Plus	Non-smartphone-based handheld fundus camera featuring a handset with a digital ophthalmoscope attachment	40-degree static field of view	6800 USD; no smartphone needed

### Photography protocol

All photographs were obtained by a single photographer who had no previous experience with fundus photography. The rationale for this was to mimic the translational practical application scenario in which a non-ophthalmologist medical technician or community health worker would use the handheld fundus cameras to conduct screening at primary care clinics or community health centers. Dilating drops (tropicamide 1% and phenylephrine 2.5%) were instilled into each eye, and after full pupillary dilation, the photographer imaged the retinas of both eyes with all three cameras in a random order. Photographs were taken in the ambient lighting conditions of the hospital ward. For the iNview and the Pictor Plus, the goal was to capture a single high-quality image of the macula, centered at the fovea and including the entire optic nerve. Imaging was repeated up to 4 times until a high-quality photograph was captured. The photographer subsequently chose the highest-quality image for photo-grading. The Pictor Plus photographs measured 1536 x 1152 pixels (72 pixels/inch) and the iNview images measured 890 x 890 pixels (72 pixels/inch). Because the narrow field of view of the Peek Retina would not permit a single image of the macula, a video (30 frames per second) of the fundus was captured under the lower-level illumination setting and converted into single-frame images by the photographer, with the final images measuring 1212 x 1978 pixels (144 pixels/inch). No post-processing of the selected images was performed. The time taken to obtain a fundus photograph was recorded for each camera for each eye. In addition, the participant reported their level of discomfort for each camera after photographs were captured for both eyes on a scale from 0 to 10, with 0 indicating no discomfort and 10 indicating the highest level of discomfort. All images were labelled with a random number identifier by the photographer in order to allow masking of the photo-graders for image analysis.

### Remote interpretation of fundus photographs

Photo-grading was performed by a tele-ophthalmology image-reading center established for this study, consisting of 8 ophthalmologists divided into 4 two-person teams. Each enrolled eye was assigned to one of the teams, and images from all three cameras were graded by the same team. Both team members independently graded each fundus image, masked both to camera type and to the grades of the other grader. A standardized form was used to document image clarity (i.e., poor, fair, good, excellent, with pre-defined categorizations based on visibility of small vessels within 1 optic disc diameter around the macula, the nerve fiber layer, and third-generation branches), coverage of the optic nerve and macula (i.e., none, partial, full), and the presence and severity of DR, diabetic macular edema, macular degeneration, and other macular pathology. [24]

DR severity was graded according to the ETDRS grading system as mild nonproliferative DR, moderate nonproliferative DR, severe nonproliferative DR, or proliferative DR. [25] Severity of macular degeneration was graded according to a clinical grading system as early AMD, intermediate AMD, or advanced AMD.^27^ A third adjudicating grader assessed all images in which the original pair of photo-graders for each team had discrepancies for their grades regarding the presence of DR, DME, or AMD. The median grade was taken from the two or three graders to create a consensus grade. Photo-graders were required to complete an online training session before beginning remote interpretation of fundus photographs in order to standardize the tele-ophthalmology image-reading procedure.

### Statistical considerations

The primary summary measure was the sensitivity and specificity of each camera for detection of DR, DME, and macular degeneration, computed relative to the presence of the respective diagnosis on the reference standard ophthalmologic examination. For the purposes of this study, the reference standard diagnosis of macular degeneration was defined as a diagnosis of age-related macular degeneration, pigment epithelial detachment, choroidal neovascularization, or polypoidal choroidal vasculopathy on ophthalmologist examination. The reference standard for DR was “any DR,” chosen because we assumed all patients with DR, even mild DR, would be referred for a full ophthalmologic examination. The intraclass correlation coefficient (ICC) was calculated from the photo-grades of the two primary graders to provide an estimate of inter-rater reliability. Diagnostic test accuracy measures and pairwise differences in these measurements between cameras were calculated with bootstrapped 95% confidence intervals, with resampling performed at the person level to account for the within-person correlation; N = 9999 replications. Analyses were performed with the statistical software R version 3.5.1 (R Project for Statistical Computing, Vienna, Austria).

### Sample size considerations

Assuming an eye-level prevalence of fundus disease of 75%, a design effect of 2 to account for within-patient correlation, and a diagnostic accuracy metric of 80%, then 240 eyes (120 participants) would provide a 95% confidence interval of ± 7.5% around the estimate of the metric.

## Results

### Enrollment

A total of 370 eyes from 185 participants were enrolled in the study, of which 355 (96%) eyes from 185 participants had a photograph available from all 3 cameras and were included in analyses. Study participants had a median age of 63 years (interquartile range [IQR] = 55 to 71) and 103 (56%) were women.

### Reference standard examination

According to the reference standard ophthalmologist examination 228 eyes (64.2%) had retinal pathology, including 102 eyes (28.7%) with DR, 71 (20.0%) with diabetic macular edema, and 89 (25.1%) with age-related macular degeneration (Table 2).

**Table 2:** Eye diseases assessed by the reference standard ophthalmologic examination, among 355 eyes.

Condition	Number
Diabetic retinopathy	102
Mild or moderate NPDR	34
Severe NPDR	13
PDR	55
Diabetic macular edema	71
Macular degeneration	89
AMD	
Early AMD	7
Intermediate AMD	7
Advanced AMD	18
Polypoidal choroidal vasculopathy	45
Choroidal neovascularization	9
Pigment epithelial detachment	3
Other retinal pathology	
Vein occlusion	32
Central serous chorioretinopathy	3
Epiretinal membrane	3
Purtscher’s-like retinopathy	2
Macular scar	2
No retinal pathology	127

Numbers do not sum to the total number of eyes because some eyes had multiple conditions.

### Camera time and discomfort

Data on test duration and discomfort was available for 364 eyes and missing for 6 eyes. The mean imaging time per eye was 43 seconds (95%CI 40–45 seconds) for the iNview, 39 seconds (95%CI 37–40) for the Peek, and 38 seconds (95%CI 37–41 seconds) for the Pictor Plus. The mean patient-reported discomfort level on a scale of 0 (least discomfort) to 10 (most discomfort) was 4.3 (95%CI 4.1–4.5) for the iNview, 2.9 (95%CI 2.7–3.1) for the Peek, and 2.7 (95%CI 2.5–2.8) for the Pictor Plus.

### Index test quality

**Fig 1:**
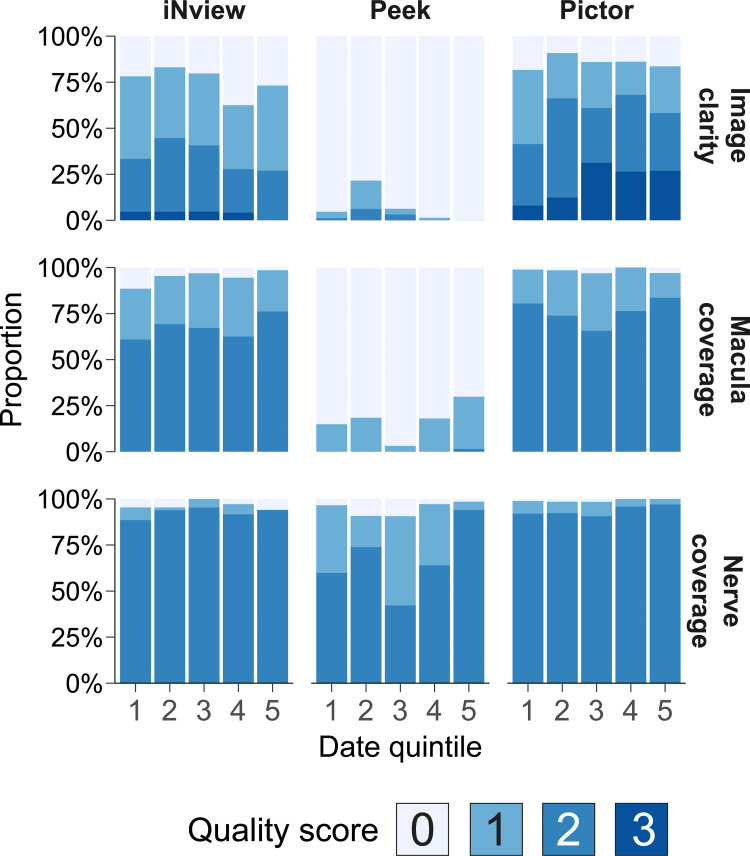
Quality assessment and learning curve of three handheld retina cameras. Each plot depicts the quality scores as a stacked bar graph over quintiles of enrollment, with quintile 1 representing the earliest enrollments. Plots are depicted stratified both by quality metric and camera. For image clarity, 0 = poor, 1 = fair, 2 = good, 3 = excellent; for coverage, 0 = absent, 1 = partly visualized, 2 = fully visualized.

**Fig 2:**
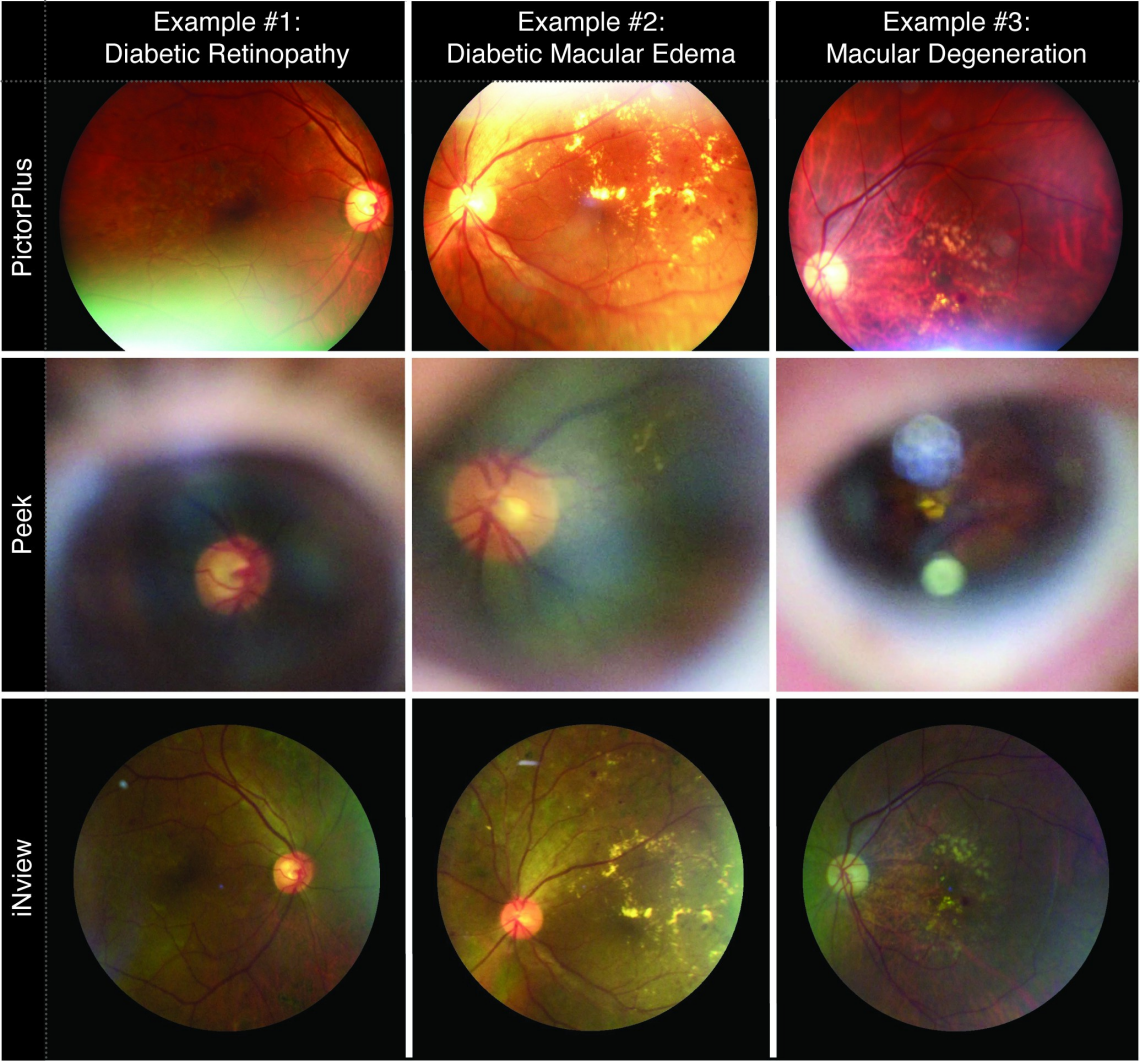
Representative images from the Pictor Plus, Peek Retina, and iNview. Fundus photos in the top, middle, and bottom rows were taken with the Pictor Plus, Peek Retina, and iNview, respectively. Each column shows photographs from the same eye, captured with different cameras; the first column depicts an eye classified by the reference standard examination as diabetic retinopathy, the second column as diabetic macular edema, and the third column as macular degeneration. Photographs are shown as captured and graded; they have been compressed in this composite image but were not otherwise manipulated with photo-editing software.

Summaries of image clarity, coverage of the optic nerve, and coverage of the macula were performed by quintile of enrollment in order to assess the learning curve for each camera (Fig 1). Over the entire study period, image clarity was deemed good or excellent for 206 (58%) Pictor Plus images, 122 (34%) iNview images, and 7 (2%) Peek images and complete coverage of the macula was achieved in 271 (76%) Pictor Plus images, 237 (67%) iNview images, and 1 (<1%) Peek image. A total of 43 (12%) Pictor Plus images, 23 (6%) iNview images, and 63 (18%) Peek Retina images were deemed ungradable. Quality did not differ markedly by age (S1 Fig). Representative images from each camera are shown for three eyes in Fig 2.

### Reproducibility of grading

Two primary graders assessed images from each of the three cameras, with a third grader adjudicating in cases of discrepant grades. Table 3 shows Cohen’s kappa statistics comparing the grades of the two primary graders for DR, DME, and macular degeneration, calculated separately for each camera. Agreement for each disease was highest for the Pictor Plus, with kappa statistics of 0.51 for AMD, 0.61 for DR, and 0.65 for DME. Agreement among photo-grading was generally higher for DR and DME than for macular degeneration, regardless of camera (Table 2).

**Table 3:** Reproducibility of photo-grades from three handheld fundus cameras.

		Kappa (95%CI)	
Camera	Diabetic retinopathy	Diabetic macular edema	Macular degeneration
iNview	0.51 (0.42–0.60)	0.56 (0.51–0.63)	0.49 (0.33–0.56)
Peek Retina	0.41 (0.27–0.52)	0.39 (0.31–0.52)	0.19 (0.10–0.34)
Pictor Plus	0.62 (0.58–0.71)	0.65 (0.59–0.75)	0.51 (0.43–0.58)

CI = bootstrapped confidence interval with person-level resampling

### Diagnostic accuracy, DR

DR was graded in 102 Pictor Plus images, 109 iNview images, and 28 Peek Retina images. Diagnostic accuracy results for detection of DR for each of the cameras are summarized in Table 4. Compared with the reference standard ophthalmologist examination, the most sensitive camera was the Pictor Plus (77%), followed by the iNview (72%), and then Peek Retina (18%). The most specific camera was the Peek Retina (96%), followed by the Pictor Plus (91%), and the iNview (86%). Sensitivity was also calculated for the subset of 47 eyes with nonproliferative DR only; this subset of eyes would be more likely to have asymptomatic disease and undergo a screening examination, while also having milder disease that would present with more subtle findings that are more difficult to detect. The sensitivities were lower for detection of only nonproliferative disease, though with a similar pattern when comparing the different cameras (sensitivities of 68% [95%CI 59–83%], 64% [95%CI 41–79%], and 19% [95%CI 10–25%], for the Pictor Plus, iNview, and Peek Retina, respectively). Exploratory analyses of test performance when the criteria for a positive index test was changed to more advanced stages of DR found considerably lower sensitivities and slightly higher specificities, regardless of whether the reference standard diagnosis was any DR (S1 Table), severe NPDR or worse (S2 Table), or PDR (S3 Table).

**Table 4:** Diagnostic Accuracy of three handheld cameras for detection of diabetic retinopathy. A positive index test was defined as a grade of any diabetic retinopathy (DR) as determined from a consensus of three photo-graders; the reference standard was the presence of any DR on ophthalmologist examination.

	Exam DR+ N = 102		Exam DR− N = 253			
Camera	Test +	Test −	Test +	Test −	Sensitivity, % (95% CI)	Specificity, % (95% CI)
iNview	73	29	36	217	71.6% (61.1–81.6%)	85.8% (80.8–90.3%)
Peek Retina	17	85	11	242	16.7% (9.0–25.6%)	95.7% (93.2–98.0%)
Pictor Plus	78	24	24	229	76.5% (67.0–85.2%)	90.5% (86.2–94.3%)

CI = bootstrapped confidence interval; DR = any diabetic retinopathy (i.e., mild nonproliferative DR or worse)

Exam = results of reference standard ophthalmologist-performed dilated fundus examination; Test = consensus results of photo-grading for each index test camera

### Diagnostic accuracy, DME

DME was diagnosed in 81 Pictor Plus images, 69 iNview images, and 24 Peek images. Estimates of diagnostic accuracy for DME are shown in Table 5; sensitivities were 72%, 55%, and 23%, and specificities 89%, 89%, and 97% for the Pictor Plus, iNview, and Peek, respectively.

**Table 5:** Diagnostic Accuracy of three handheld cameras for detection of diabetic macular edema. A positive index test was defined as a grade of any diabetic macular edema (DME) as determined from a consensus of three photo-graders; the reference standard was the presence of any DME on ophthalmologist examination.

	Exam DME + N = 71		Exam DME − N = 284			
Camera	Test +	Test −	Test +	Test −	Sensitivity, % (95% CI)	Specificity, % (95% CI)
iNview	39	32	30	254	54.9% (41.5–67.8%)	89.4% (85.5–93.1%)
Peek Retina	16	55	8	276	22.5% (11.8–34.5%)	97.2% (95.1–98.9%)
Pictor Plus	51	20	30	254	71.8% (59.6–82.9%)	89.4% (85.4–93.1%)

CI = bootstrapped confidence interval

Exam = results of reference standard ophthalmologist-performed dilated fundus examination; Test = consensus results of photo-grading for each index test camera

### Diagnostic accuracy, macular degeneration

Macular degeneration was graded in 127 Pictor Plus images, 76 iNview images, and 9 Peek Retina images. Diagnostic accuracy estimates for detection of macular degeneration (i.e., age-related macular degeneration, pigment epithelial detachment, choroidal neovascularization, or polypoidal choroidal vasculopathy on ophthalmologist examination) are summarized in Table 6 for each of the cameras. Sensitivities were estimated to be 73% for the Pictor Plus, 55% for the iNview, and 6% for Peek Retina; specificities were 77%, 90%, and 98%, respectively.

**Table 6:** Diagnostic accuracy of three handheld cameras for detection of macular degeneration. A positive index test was defined as a grade of any macular degeneration (MD) as determined from a consensus of three photo-graders; the reference standard was the presence of any findings of MD (i.e., diagnosis of age-related macular degeneration, pigment epithelial detachment, choroidal neovascularization, or polypoidal choroidal vasculopathy) on ophthalmologist examination.

	Exam MD + N = 89		Exam MD − N = 266			
Camera	Test +	Test −	Test +	Test −	Sensitivity, % (95% CI)	Specificity, % (95% CI)
iNview	49	40	27	239	55.1% (45.2–64.9%)	89.8% (85.6–93.6%)
Peek Retina	5	84	4	262	5.6% (1.2–10.8%)	98.5% (96.9–99.6%)
Pictor Plus	65	24	62	204	73.0% (64.0–81.9%)	76.7% (71.0–82.3%)

CI = bootstrapped confidence interval

Exam = results of reference standard ophthalmologist-performed dilated fundus examination; Test = consensus results of photo-grading for each index test camera

### Diagnostic accuracy, any macular pathology

Because some retinal findings might have been difficult to classify, a sensitivity analysis was performed to assess the diagnostic accuracy of detecting any retinal pathology (i.e., a grade of DR, DME, AMD, or “other macular pathology” relative to a reference standard consisting of any of the diagnoses in Table 2). This analysis found higher sensitivity than the disease-specific assessments, though the specificity was substantially reduced (Table 7).

**Table 7:** Diagnostic Accuracy of three handheld cameras for detection of any macular pathology. A positive index test was defined as a grade of any diabetic retinopathy, diabetic macular edema, macular degeneration, or other macular pathology as determined from a consensus of three photo-graders; the reference standard was the presence of any DME on ophthalmologist examination.

	Exam + N = 228		Exam − N = 127			
Camera	Test +	Test −	Test +	Test −	Sensitivity, % (95% CI)	Specificity, % (95% CI)
iNview	161	67	28	99	70.6% (64.4–76.3%)	78.0% (69.7–85.2%)
Peek Retina	37	191	2	125	16.2% (11.5–21.0%)	98.4% (95.9–100%)
Pictor Plus	189	39	48	79	82.9% (77.8–87.9%)	62.2% (53.4–71.4%)

CI = bootstrapped confidence interval

Exam = results of reference standard ophthalmologist-performed dilated fundus examination; Test = consensus results of photo-grading for each index test camera

### Predictive values

**Fig 3:**
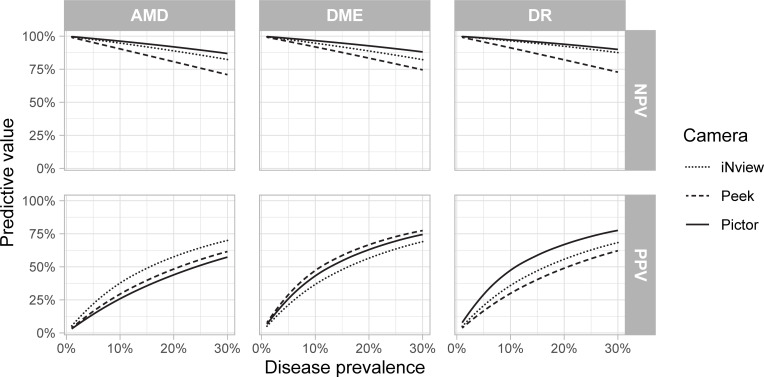
Positive and negative predictive values. Predictive values were estimated across a range of possible disease prevalences for age-related macular degeneration (AMD), diabetic macular edema (DME) and diabetic retinopathy (DR). The top row of graphs show negative predictive value (NPV) and the bottom row positive predictive value (PPV).

Positive and negative predictive values were estimated across a range of possible disease prevalences for each of the three conditions of interest (Fig 3). Negative predictive values were consistently highest for the Pictor Plus across all three conditions of interest, whereas estimates of positive predictive values were more variable.

### Comparisons

**Fig 4:**
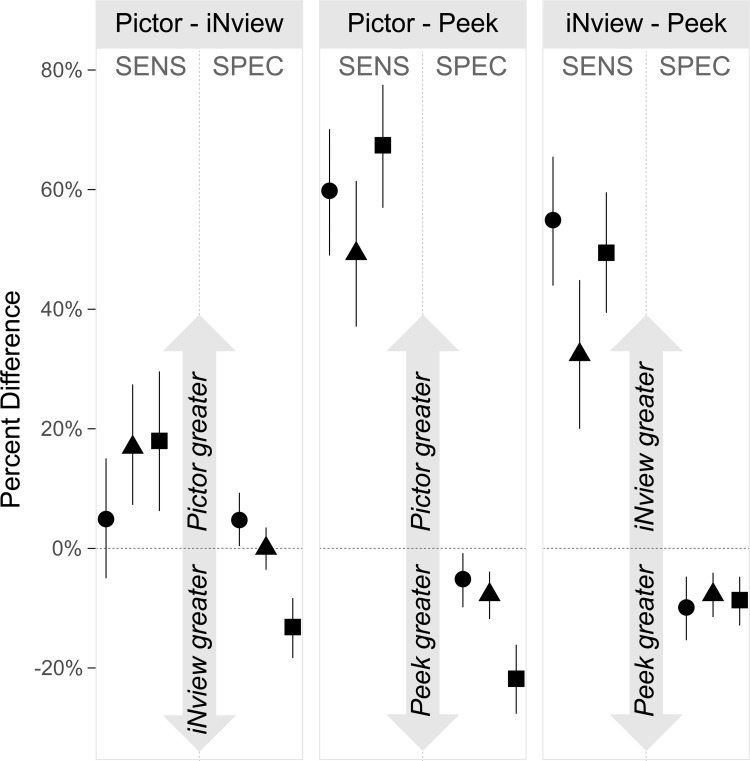
Pairwise differences in diagnostic accuracy between three handheld fundus cameras. Points represent the difference in diagnostic accuracy between two cameras, with bootstrapped 95% confidence intervals. Each panel compares the diagnostic accuracy of a different pair of cameras for diabetic retinopathy (circle), diabetic macular edema (triangle), and macular degeneration (square), with sensitivity (SENS) on the left side of the panel and specificity (SPEC) on the right.

Fig 4 shows pairwise differences in sensitivity and specificity for the three cameras. The Pictor Plus and iNview were each statistically significantly more sensitive and less specific than Peek (i.e., the 95% confidence intervals did not include zero for any comparison) for all three eye diseases of interest. The Pictor Plus was more sensitive than the iNview for all three diseases, though the differences were of lower magnitude than the pairwise comparisons with Peek Retina, and the difference was not statistically significant for the DR outcome. Comparisons of specificity between the Pictor Plus and iNview were variable, with the Pictor Plus significantly more specific for DR and the iNview more specific for macular degeneration.

## Discussion

This study compared the image quality, diagnostic accuracy, and user experience of three commercially available handheld fundus cameras when used to photograph the posterior segment through a dilated pupil. The Pictor Plus achieved the highest scores for image clarity and coverage of the macula and optic nerve, although the iNview also scored highly on quality metrics. The sensitivity of the Peek Retina attachment was lower than the other two cameras for detecting DR, DME, or AMD. Specificity was relatively high for all three tested cameras, with generally higher estimates for diagnosis of DR and DME relative to macular degeneration. In terms of the user experience, imaging time was similar between all three cameras, and participant-reported discomfort was slightly higher for the iNview relative to the other two cameras.

Quality was assessed from the images by judging clarity and coverage of the optic nerve and macula. A main finding was that the clarity assessments varied quite a bit between the three cameras, with the Pictor Plus having the clearest images and Peek Retina the least clear images. This is likely attributable to the built-in construction of the camera and optics of the Pictor Plus, while the smartphone-based cameras require manual assembly of the optical system. The Peek Retina in particular has a simple optical system, which limited the field of view of images and necessitated multiple scans across the macula in attempts to ensure adequate coverage. Despite these attempts, the macula coverage was poor for the Peek Retina, which likely contributed to a lower score for quality of photographs. The Pictor Plus had a tendency to overexpose photographs with its automatic image-capture settings, while the Peek Retina images frequently were underexposed, which reduced the ability to discriminate retinal findings (e.g., Fig 2). While it is possible that post-processing could have enhanced the quality of some photographs, the decision was made to not pursue post-processing of any images in this study, as such photo-editing would likely not be practical or feasible in a real-world setting of retinal disease screening.

Each of the three cameras had varying advantages and disadvantages with regards to the patient-photographer interface and user performance. Overall, the median time spent capturing a fundus image for each eye ranged between 30 to 40 seconds for all of the cameras, which is satisfactorily fast enough for screening purposes in a practical setting. According to the study photographer, images were easiest to capture with the Pictor Plus, largely because of its autofocus feature. The iNview and Peek Retina, in contrast, required increased time for manual manipulation of the device to maximize the chances of capturing an in-focus retinal image, with the Peek Retina requiring more photography skills to adequately capture the entire posterior pole of the fundus due to its small field of view. The Peek Retina also required more post-imaging time than the other cameras since the photographer had to scrub through the captured video to select multiple images that provided visualization of different parts of the fundus.

Participants reported more discomfort with the iNview than the other cameras, likely due to constant illumination from the native smartphone LED light source during image capture. The Peek Retina also required constant illumination for video capture, but offered two levels of brightness, both of which were less bright than the iNview. One feature of the Pictor Plus that may have added to patient comfort was its use of an infrared light source for focusing before image capture. Neither of the smartphone-based devices used infrared light, perhaps because some smartphone cameras have filters that block infrared light.

The Pictor Plus produced images that provided the most reproducible grading and by the majority of metrics, had the most optimal balance of sensitivity, specificity, and predictive value. For example, the Pictor Plus had a considerably higher positive predictive value (i.e., fewer false positives) than the other two fundus cameras across a range of plausible DR prevalences, while also having a higher negative predictive value (i.e., fewer false negatives; Fig 3). It is worth noting that even the Pictor Plus had only an approximately 50% positive predictive value if the underlying population prevalence of DR was 10%, suggesting that the best-use case scenario for such a handheld camera might be for screening populations expected to be have higher risk of disease (e.g., patients with advanced diabetes). Images from the iNview did not lag significantly behind those from the Pictor Plus in terms of quality and diagnostic accuracy, while offering the advantage of a significantly lower cost. In contrast, the Peek Retina had considerably lower sensitivity, likely due to its narrower field of view.

Other features of the cameras may be important besides diagnostic accuracy. For example, the iNview uses an integrated smartphone-based application for securely uploading, storing, and sharing encrypted images. The iNView also offers a lightweight attachment design that, unlike the Peek Retina and Pictor Plus, does not require separate charging of the device in addition to the smartphone. The Pictor Plus is marketed as a nonmydriatic camera, with a previous study noting that it captured good quality nonmydriatic photographs of the optic nerve; however, we did not assess nonmydriatic imaging for any of the cameras in the present study as the Peek Retina and iNview both required pupillary dilation for effective fundus image capture. [19]

Various limitations of this study should be noted. First, the reference standard for assessing diagnostic accuracy was a dilated funduscopic examination by an ophthalmologist, which may be less sensitive than high-quality fundus photography for screening purposes. [26] Using a conventional table-top fundus camera as the reference standard would have offered some advantages, such as the ability to mask the outcome assessors and to audit photographs at a later time. However, we chose not to subject the participants in this study to a fourth photo modality in consideration of their time and discomfort, reasoning that the clinical examination, which would take place regardless of study participation, would be an adequate reference standard. Second, because the field of view differed for each of the cameras (50 degrees for the iNview, 20 degrees for the Peek Retina, 40 degrees for the Pictor Plus), it was difficult to determine whether the field of view or the image clarity was more important for diagnostic accuracy. Third, the patient population in this study was enriched for retinal disease requiring treatment and thus, it is uncertain whether these results will be generalizable to other settings in populations with less advanced disease. Finally, this study used three specific handheld fundus cameras and imaged through a dilated pupil, and thus its generalizability to other fundus cameras or for undilated pupils is not clear. Nonetheless, this study was carried out rigorously and thus provides a variety of useful benchmarks to gauge the diagnostic performance of other devices. Despite these limitations, the present study is valuable in that it provides an estimate of the best performance that these handheld fundus cameras may be expected to demonstrate when used in a clinical setting for retinal disease screening.

Tele-ophthalmology and screening programs will only increase in frequency, scope, and necessity in the years to come. Comparative studies like the present study help tele-medicine program managers better understand the capabilities of a diagnostic modality in general (e.g., handheld fundus cameras) as well as the capabilities of specific instruments (e.g., the three fundus cameras analyzed in this study). Future comparative studies in real-world settings using less specialized photo-graders or even novel technologies such as automated image analysis will be noteworthy for determining optimal practices for tele-ophthalmology screening programs that will be valuable in advancing health care in both developed and developing countries worldwide.

## Supporting information

S1 Data Study data. (CSV)

S1 Fig Quality assessment and learning curve of three handheld retina cameras, stratified by age. Quality scores are shown stratified by age, with younger participants (ages < 63 years) in the left panel and older participants (ages ≥ 63 years) in the right panel. Each plot depicts the quality scores as a stacked bar graph over quintiles of enrollment, with quintile 1 representing the earliest enrollments. Plots are depicted stratified both by quality metric and camera. For image clarity, 0 = poor, 1 = fair, 2 = good, 3 = excellent; for coverage, 0 = absent, 1 = partly visualized, 2 = fully visualized.
(EPS)

S1 Table Diagnostic Accuracy of three handheld cameras for detection of any DR. A positive index test was determined from a consensus of three photo-graders, and defined in three ways: first, as a grade of any diabetic retinopathy (DR), second, as a grade of severe nonproliferative DR (NPDR) or proliferative DR (PDR), and third, as a grade of PDR. The reference standard was the presence of any DR on ophthalmologist examination.
(DOCX)

S2 Table Diagnostic Accuracy of three handheld cameras for detection of severe nonproliferative diabetic retinopathy or worse. A positive index test was determined from a consensus of three photo-graders, and defined in three ways: first, as a grade of any diabetic retinopathy (DR), second, as a grade of severe nonproliferative DR (NPDR) or proliferative DR (PDR), and third, as a grade of PDR. The reference standard was the presence of severe nonproliferative DR or proliferative DR on ophthalmologist examination.
(DOCX)

S3 Table Diagnostic Accuracy of three handheld cameras for detection of proliferative diabetic retinopathy. A positive index test was determined from a consensus of three photo-graders, and defined in three ways: first, as a grade of any diabetic retinopathy (DR), second, as a grade of severe nonproliferative DR (NPDR) or proliferative DR (PDR), and third, as a grade of PDR. The reference standard was the presence of proliferative DR on ophthalmologist examination.
(DOCX)
