# Supplementary material for: Diagnostic accuracy of handheld fundus photography: A comparative study of three commercially available cameras
Source: PLOS Digit Health. 2022 Nov 2;1(11):e0000131. doi: 10.1371/journal.pdig.0000131 (PMC9931246; doi:10.1371/journal.pdig.0000131)
Supplement: S1 Table — A positive index test was determined from a consensus of three photo-graders, and defined in three ways: first, as a grade of any diabetic retinopathy (DR), second, as a grade of severe nonproliferative DR (NPDR) or proliferative DR (PDR), and third, as a grade of PDR. The reference standard was the presence of any DR on ophthalmologist examination. (DOCX) [file pdig.0000131.s003.docx]

**S1 Table. Diagnostic Accuracy of three handheld cameras for detection of any DR.** A positive index test was determined from a consensus of three photo-graders, and defined in three ways: first, as a grade of any diabetic retinopathy (DR), second, as a grade of severe nonproliferative DR (NPDR) or proliferative DR (PDR), and third, as a grade of PDR. The reference standard was the presence of any DR on ophthalmologist examination.

|  | Exam any DR +  N=102 | |  | Exam any DR −  N=253 | |  |  |
| --- | --- | --- | --- | --- | --- | --- | --- |
| Camera | Test + | Test − |  | Test + | Test − | Sensitivity, % (95% CI) | Specificity, % (95% CI) |
| Index test: Any DR |  |  |  |  |  |  |  |
| iNview | 73 | 29 |  | 36 | 217 | 71.6% (61.1-81.6%) | 85.8% (80.8-90.3%) |
| Peek Retina | 17 | 85 |  | 11 | 242 | 16.7% (9.0-25.6%) | 95.7% (93.2-98.0%) |
| Pictor Plus | 78 | 24 |  | 24 | 229 | 76.5% (67.0-85.2%) | 90.5% (86.2-94.3%) |
| Index test: ≥ Moderate NPDR |  |  |  |  |  |  |  |
| iNview | 54 | 48 |  | 31 | 222 | 52.9% (42.1-63.9%) | 87.7% (83.1-92.0%) |
| Peek Retina | 11 | 91 |  | 8 | 245 | 10.8% (4.9-18.1%) | 96.8% (94.6-98.8%) |
| Pictor Plus | 60 | 42 |  | 21 | 232 | 58.5% (47.9-69.6%) | 91.7% (87.7-95.3%) |
| Index test: ≥ Severe NPDR |  |  |  |  |  |  |  |
| iNview | 16 | 86 |  | 15 | 238 | 15.7% (8.5-23.8%) | 94.1% (91.1-96.8%) |
| Peek Retina | 5 | 97 |  | 7 | 246 | 4.9% (1.0-9.5%) | 97.2% (95.1-99.1%) |
| Pictor Plus | 24 | 78 |  | 14 | 239 | 23.5% (14.4-33.3%) | 94.4% (91.4-97.2%) |
| Index test: PDR |  |  |  |  |  |  |  |
| iNview | 9 | 93 |  | 10 | 243 | 8.8% (3.9-14.6%) | 96.0% (93.6-98.2%) |
| Peek Retina | 3 | 99 |  | 6 | 247 | 2.9% (0-6.6%) | 97.6% (95.6-99.2%) |
| Pictor Plus | 10 | 92 |  | 8 | 245 | 9.8% (4.4-15.7%) | 96.8% (94.6-98.8%) |

CI=bootstrapped confidence interval; Exam=results of reference standard ophthalmologist-performed dilated fundus examination; Index test=consensus results of photo-grading
