# Supplementary material for: Diagnostic accuracy of handheld fundus photography: A comparative study of three commercially available cameras
Source: PLOS Digit Health. 2022 Nov 2;1(11):e0000131. doi: 10.1371/journal.pdig.0000131 (PMC9931246; doi:10.1371/journal.pdig.0000131)
Supplement: S2 Table — A positive index test was determined from a consensus of three photo-graders, and defined in three ways: first, as a grade of any diabetic retinopathy (DR), second, as a grade of severe nonproliferative DR (NPDR) or proliferative DR (PDR), and third, as a grade of PDR. The reference standard was the presence of severe nonproliferative DR or proliferative DR on ophthalmologist examination. (DOCX) [file pdig.0000131.s004.docx]

**S2 Table. Diagnostic Accuracy of three handheld cameras for detection of severe nonproliferative diabetic retinopathy or worse.** A positive index test was determined from a consensus of three photo-graders, and defined in three ways: first, as a grade of any diabetic retinopathy (DR), second, as a grade of severe nonproliferative DR (NPDR) or proliferative DR (PDR), and third, as a grade of PDR. The reference standard was the presence of severe nonproliferative DR or proliferative DR on ophthalmologist examination.

|  | Exam severe NPDR or worse +  N=68 | |  | Exam severe  NPDR or worse −  N=287 | |  |  |
| --- | --- | --- | --- | --- | --- | --- | --- |
| Camera | Test + | Test − |  | Test + | Test − | Sensitivity, % (95% CI) | Specificity, % (95% CI) |
| Index test: Any DR |  |  |  |  |  |  |  |
| iNview | 56 | 12 |  | 53 | 234 | 82.4% (71.7-91.7%) | 81.5% (76.2-86.7%) |
| Peek Retina | 11 | 57 |  | 17 | 270 | 16.2% (7.6-25.7%) | 94.1% (91.0-96.9%) |
| Pictor Plus | 58 | 10 |  | 44 | 243 | 85.3% (75.4-93.8%) | 84.7% (79.6-89.5%) |
| Index test: ≥ Moderate NPDR |  |  |  |  |  |  |  |
| iNview | 47 | 21 |  | 38 | 249 | 69.1% (56.5-81.1%) | 86.8% (82.1-91.1%) |
| Peek Retina | 7 | 61 |  | 12 | 275 | 10.3% (3.9-17.9%) | 95.8% (93.2-98.2%) |
| Pictor Plus | 50 | 18 |  | 31 | 256 | 73.5% (62.1-84.1%) | 89.2% (85.0-93.0%) |
| Index test: ≥ Severe NPDR |  |  |  |  |  |  |  |
| iNview | 15 | 53 |  | 16 | 271 | 22.1% (71.7-91.7%) | 94.4% (91.8-97.0%) |
| Peek Retina | 5 | 63 |  | 7 | 280 | 7.4% (1.7-14.1%) | 97.6% (95.7-99.3%) |
| Pictor Plus | 21 | 47 |  | 17 | 270 | 30.9% (18.6-44.6%) | 94.1% (91.1-96.8%) |
| Index test: PDR |  |  |  |  |  |  |  |
| iNview | 8 | 60 |  | 11 | 276 | 11.8% (5.0-19.7%) | 96.2% (93.9-98.2%) |
| Peek Retina | 3 | 65 |  | 6 | 281 | 4.4% (0-9.7%) | 97.9% (96.1-99.3%) |
| Pictor Plus | 9 | 59 |  | 9 | 278 | 13.2% (5.7-21.7%) | 96.9% (94.8-98.7%) |

CI=bootstrapped confidence interval; Exam=results of reference standard ophthalmologist-performed dilated fundus examination; Index test=consensus results of photo-grading
