# Supplementary material for: Diagnostic accuracy of handheld fundus photography: A comparative study of three commercially available cameras
Source: PLOS Digit Health. 2022 Nov 2;1(11):e0000131. doi: 10.1371/journal.pdig.0000131 (PMC9931246; doi:10.1371/journal.pdig.0000131)
Supplement: S3 Table — A positive index test was determined from a consensus of three photo-graders, and defined in three ways: first, as a grade of any diabetic retinopathy (DR), second, as a grade of severe nonproliferative DR (NPDR) or proliferative DR (PDR), and third, as a grade of PDR. The reference standard was the presence of proliferative DR on ophthalmologist examination. (DOCX) [file pdig.0000131.s005.docx]

**S3 Table. Diagnostic Accuracy of three handheld cameras for detection of proliferative diabetic retinopathy.** A positive index test was determined from a consensus of three photo-graders, and defined in three ways: first, as a grade of any diabetic retinopathy (DR), second, as a grade of severe nonproliferative DR (NPDR) or proliferative DR (PDR), and third, as a grade of PDR. The reference standard was the presence of proliferative DR on ophthalmologist examination.

|  | Exam PDR +  N=55 | |  | Exam PDR −  N=300 | |  |  |
| --- | --- | --- | --- | --- | --- | --- | --- |
| Camera | Test + | Test − |  | Test + | Test − | Sensitivity, % (95% CI) | Specificity, % (95% CI) |
| Index test: Any DR |  |  |  |  |  |  |  |
| iNview | 43 | 12 |  | 66 | 234 | 78.2% (65.5-89.6%) | 78.0% (72.2-83.5%) |
| Peek Retina | 8 | 47 |  | 20 | 280 | 14.5% (5.3-25.0%) | 93.3% (90.2-96.1%) |
| Pictor Plus | 46 | 9 |  | 56 | 244 | 83.6% (72.1-93.4%) | 81.3% (75.9-86.4%) |
| Index test: ≥ Moderate NPDR |  |  |  |  |  |  |  |
| iNview | 37 | 18 |  | 48 | 252 | 67.3% (52.9-81.3%) | 84.0% (79.1-88.5%) |
| Peek Retina | 4 | 51 |  | 15 | 285 | 7.3% (1.7-14.5%) | 95.0% (92.2-97.4%) |
| Pictor Plus | 39 | 16 |  | 42 | 258 | 70.9% (57.9-83.1%) | 86.0% (81.4-90.4%) |
| Index test: ≥ Severe NPDR |  |  |  |  |  |  |  |
| iNview | 9 | 46 |  | 22 | 278 | 16.4% (7.7-26.4%) | 92.7% (89.5-95.6%) |
| Peek Retina | 3 | 52 |  | 9 | 291 | 5.5% (0-11.9%) | 97.0% (94.9-98.7%) |
| Pictor Plus | 16 | 39 |  | 22 | 278 | 29.1% (15.6-44.2%) | 92.7% (89.3-95.7%) |
| Index test: PDR |  |  |  |  |  |  |  |
| iNview | 8 | 47 |  | 11 | 289 | 14.5% (6.1-24.5%) | 96.3% (94.1-98.3%) |
| Peek Retina | 3 | 52 |  | 6 | 294 | 5.5% (0-11.8%) | 98.0% (96.4-99.4%) |
| Pictor Plus | 7 | 48 |  | 11 | 289 | 12.7% (4.9-21.7%) | 96.3% (94.1-98.3%) |

CI=bootstrapped confidence interval; Exam=results of reference standard ophthalmologist-performed dilated fundus examination; Index test=consensus results of photo-grading
